# Synthesis and crystal structure of dipotassium nickel polyphosphate

**DOI:** 10.1107/S2056989025002221

**Published:** 2025-03-19

**Authors:** El Waleda Moustapha Thiam, Kalidou Mamadou Ba, Aichata Yaya Kane, Ibrahima Elhadji Thiam, Nicolas Claiser, Mohamed Souhassou, Aliou Hamady Barry, Mohamed Gaye

**Affiliations:** a Unité de Recherche en Chimie des Matériaux, Département de Chimie, Faculté des Sciences et Techniques, Université de Nouakchott, Mauritania; b Département de Chimie Faculté des Sciences et Techniques Université Cheik Anta Diop Dakar Senegal; c CNRS Laboratoire CRM2 UMR CNRS 7036 Université de Lorraine, boulevard des aiguillettes BP 70239 Vandoeuvre-lès-Nancy 54506 France; University of Missouri-Columbia, USA

**Keywords:** crystal structure, phosphate, potassium, nickel, octa­hedral, penta­gonal

## Abstract

Single crystals of K_2_Ni(PO_3_)_4_ were obtained by solid-state reaction. The structure consists of infinite zigzag polyphosphate chains, running along the *c*-axis direction, linked by Ni^2+^ ions and delimiting large tunnels in which the K^+^ ions are located. Ni^2+^ ions form slightly distorted NiO_6_ octa­hedra and the coordination numbers of the independent potassium cations are 8 and 10.

## Abstract 2

The structure consists of infinite zigzag polyphosphate chains, running along the *c*-axis direction, linked by Ni^2+^ ions and delimiting large tunnels in which the K^+^ ions are located. Ni^2+^ ions form slightly distorted NiO_6_ octa­hedra and the coordination numbers of the independent potassium cations are 8 and 10.

## 1. Chemical context

Transition-metal oxides (Fe, Co, or Ni) in melted phosphate systems of alkali metals (*M*_2_O–P_2_O_5_, *M* = Li, Na, or K) are widely studied (Pontchara & Durif, 1974 ▸; Litvin & Masloboev, 1989 ▸; Panahandeh & Jung, 2003 ▸; Moutataouia *et al.*, 2014 ▸; Ouaatta *et al.*, 2019 ▸). These materials present various and inter­esting properties and applications, such as catalysts (Moffat, 1978 ▸), ferroelectric and/or magnetic materials (Lazoryak *et al.*, 2004 ▸; Hatert *et al.*, 2004 ▸; Essehli *et al.*, 2015 ▸) and ion-conduction properties (La Parola *et al.*, 2018 ▸; Orikasa *et al.*, 2016 ▸; Daidouh *et al.*, 1999 ▸). Some of these specific properties, such as catalytic activity, are similar to those found in the phosphate systems themselves (Kapshuk *et al.*, 2000 ▸). These compounds have been characterized by several physico-chemical and structural methods. Among these studies, some have been devoted to nickel-based phosphates associated with alkali metals, such as the polyphosphates *M*Ni(PO_3_)_3_ (*M* = Li, Na, or K; Kapshuk *et al.*, 2000 ▸) and NiCs_4_(PO_3_)_6_ (Sbai *et al.*, 2004 ▸). For all these samples, the study of their structural characteristics is essential for understanding most of the physical properties (Fischer *et al.*, 1994 ▸). The title polyphos­phate, K_2_Ni(PO_3_)_4_, designated as (**1**), was obtained in the quest to synthesize new condensed phosphates appearing in the *A*_2_O–*M*O–*Ln*O_3_–P_2_O_5_ quaternary system (*A*: an alkali metal, *M*: transition metal divalent cation, *Ln*: lanthanide or *Y* metal). This compound has been observed in the diagram Ni(PO_3_)_2_–KPO_3_ (Pontchara & Durif, 1974 ▸) but, to our knowledge, its crystal structure has not yet been reported. We report herein on its synthesis and structural characterization by single crystal X-ray diffraction.

## 2. Structural commentary

**Figure 1:**
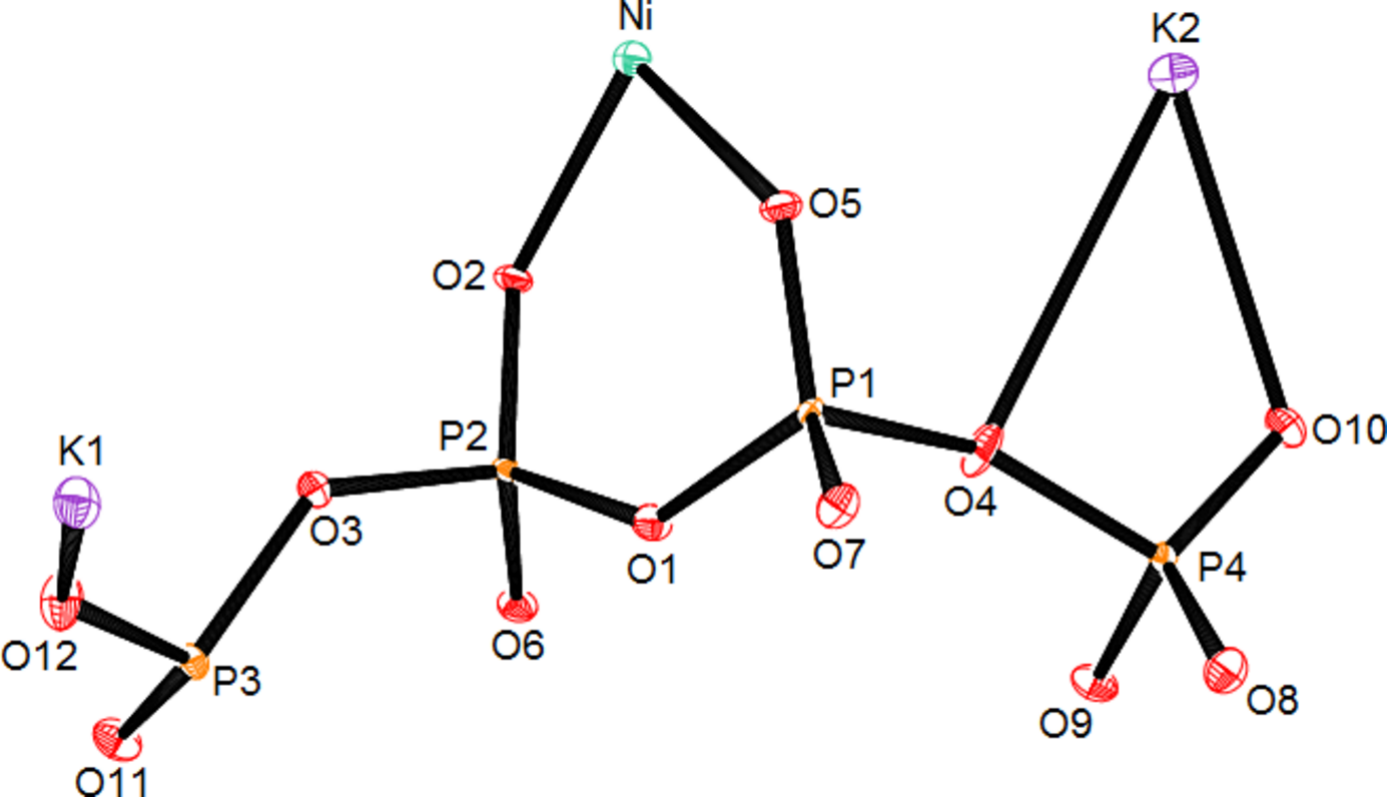
The asymmetric unit of K_2_Ni(PO_3_)_4_.

**Figure 2:**
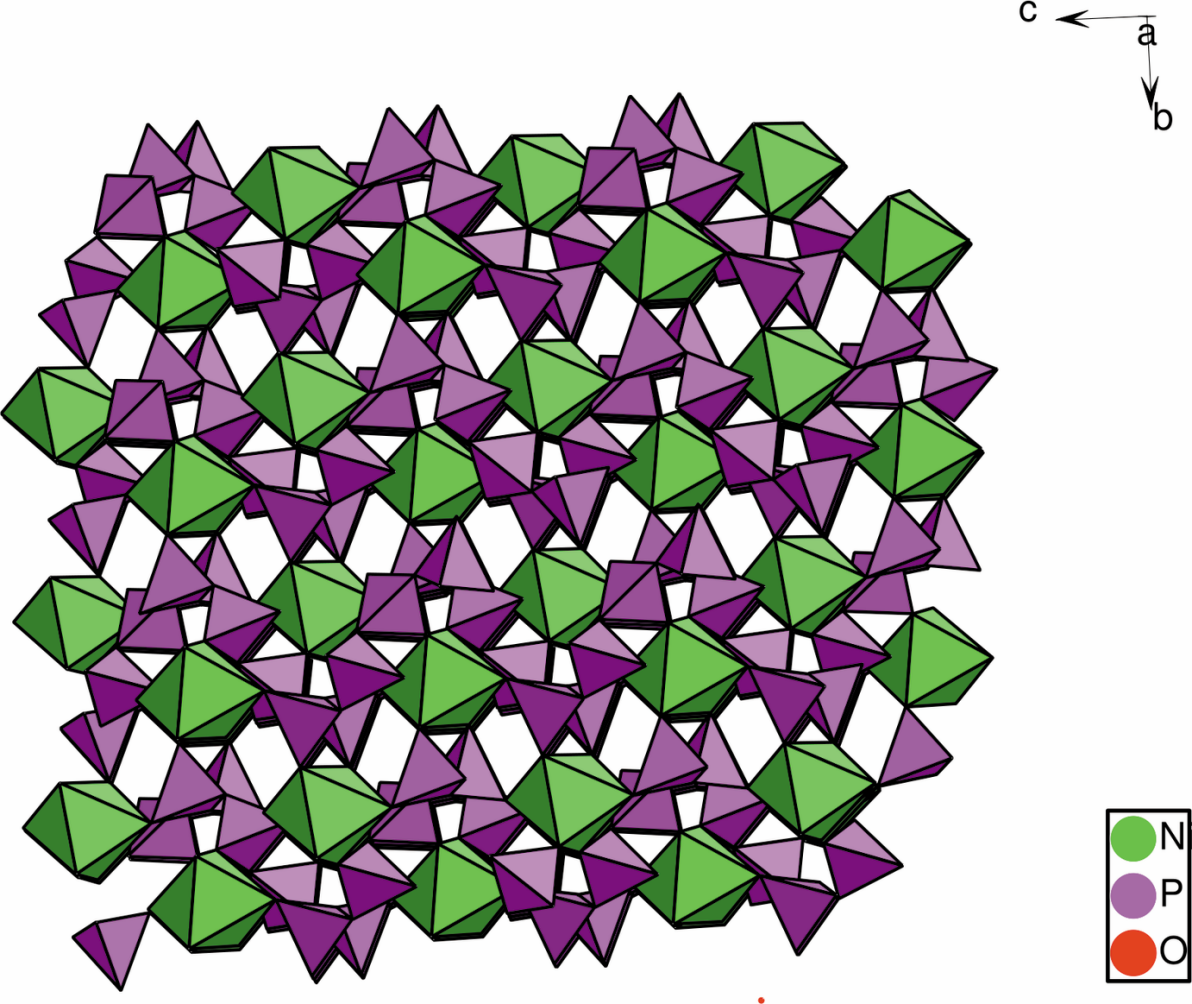
Projection of the K_2_Ni(PO_3_)_4_ structure along [100].

**Figure 3:**
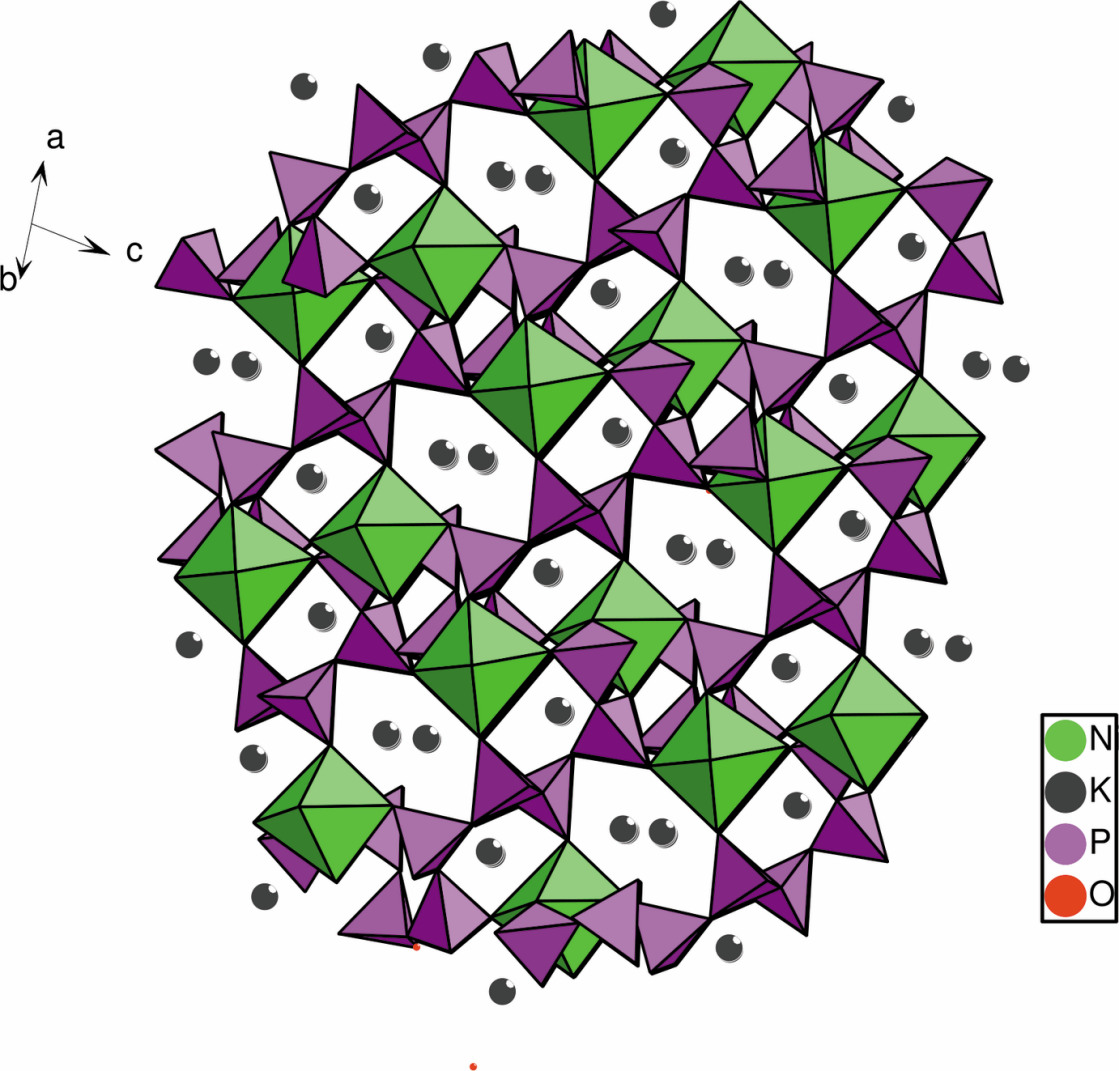
Polyhedral representation of the structure of K_2_Ni(PO_3_)_4_ showing the tunnels in which the K^+^ cations are located.

The title compound, (**1**), crystallizes in the non-centrosymmetric monoclinic space group *Cc*. The asymmetric unit contains 19 atoms corresponding to the chemical formula K_2_Ni(PO_3_)_4_, as shown in Fig. 1 ▸. The structure is based on infinite zigzag polyphosphate chains running almost along the *c*-axis direction and linked by NiO_6_ octa­hedra (Fig. 2 ▸). Each NiO_6_ octa­hedron shares corners with six different PO_4_ tetra­hedra belonging to three polyphosphate chains. All the terminal O atoms of the PO_4_ tetra­hedra in the polyphosphate chains inter­act with the Ni and K atoms. Such an arrangement creates a three-dimensional framework that delimits large hexa­gonal and penta­gonal tunnels in which the K^+^ ions are located (Fig. 3 ▸). The NiO_6_ octa­hedra are slightly distorted, with Ni—O distances ranging from 2.017 (2) to 2.167 (2) Å and the O—Ni—O angles from 82.52 (6)° to 173.51 (6)°. In the four PO_4_ tetra­hedra, the equatorial and apical distances P—OE and P—OL, respectively, range from 1.469 (2) to 1.493 (2) Å for P—OE and 1.578 (1) to 1.602 (1)Å for P—OL and the O—P—O angles range from 100.37 (10) to 120.76 (10)°. The unit cell contains two crystallographically non-equivalent K atoms (K1 and K2) both located in large hexa­gonal and penta­gonal tunnels (Fig. 3 ▸). The coordination number (CN) is 8 for K1 and 10 for K2. The K—O inter­actions range from 2.582 (2) and 3.111 (2) Å for K1 (mean distance: 2.841 Å) and 2.761 (2) to 3.414 (2) Å for K2 (mean distance: 3.041 Å)

## 3. Database survey

A search in the Cambridge Structural Database (Version 5.43, November 2021; Groom *et al.*, 2016 ▸) revealed about a dozen alkaline nickel-based phosphates: TlNi_4_(PO_4_)_3_, Tl_4_Ni_7_(PO_4_)_6_ and Tl_2_Ni_4_(P_2_O_7_)(PO_4_)_2_ (Panahandeh & Jung, 2003 ▸), KNi_3_(PO_4_)P_2_O_7_ (Moutataouia *et al.*, 2014 ▸), K_2_Ni_4_(PO_4_)_2_(P_2_O_7_) (Palkina *et al.*, 1980 ▸), K_2_NiP_2_O_7_ (El Maadi *et al.*, 1995 ▸), *AM*_4_(PO_4_)_3_ (*A* = Na, K, Rb; *M* = Ni, Mn) (Daidouh *et al.*, 1999 ▸), *M*Ni(PO_3_)_3_ (*M* = Na or K) (Kapshuk *et al.*, 2000 ▸), KNiPO_4_ (Fischer *et al.*, 1994 ▸), NiCs_4_(PO_3_)_6_ and NiK_4_(P_3_O_9_)_2_ (Sbai *et al.*, 2004 ▸). As in the case of the title compound, the structures of *M*Ni(PO_3_)_3_ (*M* = Na or K) polyphosphates are based on infinite zigzag polyphosphate chains, linked by Ni^2+^ ions in octa­hedral coordination and delimiting tunnels in which the alkali ions are located (Fig. 3 ▸). The metal atoms (*M*) and nickel atoms form infinite ⋯Ni–*M*–Ni–*M*⋯ columns of polyhedra sharing edges and alternating with the polyphos­phate chains. In the NaNi(PO_3_)_3_ structure (Kapshuk *et al.*, 2000 ▸), the polyphosphate chains run along the *a*-axis direction, and the Na atom exhibits a distorted octa­hedral environment whereas in KNi(PO_3_)_3_, the polyphosphate chains are run in the same direction as in the title polyphos­phate (*c*-axis) and the coordination polyhedron of the K atom is a distorted tricapped trigonal prism (CN = 9), unlike in the title polyphosphate where the K atoms exhibit two different coordin­ation numbers (8 and 10). In the other alkaline nickel-based phosphates TlNi_4_(PO_4_)_3_, Tl_4_Ni_7_(PO_4_)_6_, Tl_2_Ni_4_(P_2_O_7_)(PO_4_)_2_, KNi_3_(PO_4_)P_2_O_7_, K_2_Ni_4_(PO_4_)_2_(P_2_O_7_), K_2_NiP_2_O_7_, *A*M_4_(PO_4_)_3_ (*A* = Na, K, Rb; *M* = Ni, Mn); the structural arrangements are markedly different from that of the title compound being based on alkali and Ni polyhedra sharing edges and forming chains that are linked together by isolated PO_4_ and/or diphosphate (P_2_O_7_) groups. In addition, the coordination polyhedron of the Ni atom is not always octa­hedral. For instance, it is 7-coordinated in TlNi_4_(PO_4_)_3_, 5-coordinated in KNi_4_(PO_4_)_3_ and has an unusual coordination of only four oxygen atoms in a distorted tetra­hedron in Tl_2_Ni_4_(P_2_O_7_)(PO_4_)_2_. The coordination numbers of the metal atoms (Tl and K) in these phosphates range from 6 to 12. Thallium nickel phosphate, Tl_2_Ni_4_(P_2_O_7_)(PO_4_)_2_, adopts the K_2_Ni_4_(PO_4_)_2_(P_2_O_7_) structure, and the environments of the alkali and nickel atoms are nearly identical.

## 4. Synthesis and crystallization

Single crystals of K_2_Ni(PO_3_)_4_ were prepared by solid-state reaction. A mixture of the reagents K_2_CO_3_, NiCl_2_·6H_2_O, NH_4_H_2_PO_4_ and La_2_O_3_ in a molar ratio of K:Ni:P:La of 0.4:0.05:1:0.02 was placed in a porcelain crucible. The reaction mixture was then calcined at 623 K for 1 h and gradually heated to 823 K. Maintained at this temperature for 72 h, the reaction mixture then underwent slow cooling at a rate of 1 K h^−1^ to 773 K and then to room temperature with furnace inertia. The crystals obtained were recovered after washing with boiling water.

## 5. Refinement

Crystal data, data collection and structure refinement details are summarized in Table 1 ▸. Solving and refinement tests of the structure were carried out in both the centrosymmetric and non-centrosymmetric space groups *C*2/*c* and *Cc*. Better results with a much convergent refinement were obtained with the non-centrosymmetric model. The use of the TWIN refinement mode made the refinement results significantly improved. No Extinction correction was applied. The residual maximum and minimum electron density peaks are located 0.13 Å from P1 and 0.36 Å from Ni1, respectively. However, the minimum density observed in the vicinity of a nickel atom is rather largely negative (−2.6 e Å^−3^) indicating probably that the absorption correction applied was not optimal.

## Supplementary Material

Crystal structure: contains datablock(s) I. DOI: 10.1107/S2056989025002221/ev2014sup1.cif

Structure factors: contains datablock(s) I. DOI: 10.1107/S2056989025002221/ev2014Isup2.hkl

CCDC reference: 2430654

Additional supporting information:  crystallographic information; 3D view; checkCIF report

## Figures and Tables

**Table 1:** Experimental details

Crystal data	
Chemical formula	K_2_Ni(PO_3_)_4_
*M*_r_	452.79
Crystal system, space group	Monoclinic, *Cc*
Temperature (K)	293
*a*, *b*, *c* (Å)	11.07179 (16), 12.50386 (16), 7.53969 (11)
β (°)	103.2349 (14)
*V* (Å^3^)	1016.07 (3)
*Z*	4
Radiation type	Mo *K*α
μ (mm^−1^)	3.43
Crystal size (mm)	0.12 × 0.08 × 0.07

Data collection	
Diffractometer	SuperNova
Absorption correction	Multi-scan (*CrysAlis PRO*; Rigaku OD, 2022 ▸)
*T*_min_, *T*_max_	0.509, 0.710
No. of measured, independent and observed [*I* > 2σ(*I*)] reflections	33158, 8076, 7869
*R*_int_	0.035
(sin θ/λ)_max_ (Å^−1^)	0.999

Refinement	
*R*[*F*^2^ > 2σ(*F*^2^)], *wR*(*F*^2^), *S*	0.031, 0.088, 1.07
No. of reflections	8076
No. of parameters	173
No. of restraints	2
Δρ_max_, Δρ_min_ (e Å^−3^)	0.83, −2.85
Absolute structure	Refined as an inversion twin
Absolute structure parameter	0.539 (9)

Computer programs: *CrysAlis PRO* (Rigaku OD, 2022 ▸), *SHELXT* (Sheldrick, 2015*a* ▸), *SHELXL* (Sheldrick, 2015*b* ▸), *ORTEP-3 for Windows* (Farrugia, 2012 ▸), *DIAMOND* (Brandenburg, 2006 ▸) and *publCIF* (Westrip, 2010 ▸).

## supplementary crystallographic information

### Dipotassium nickel polyphosphate Crystal data

**Table:**

K_2_Ni(PO_3_)_4_	*F*(000) = 888
*M**_r_* = 452.79	*D*_x_ = 2.960 Mg m^−^^3^
Monoclinic, *C**c*	Mo *K*α radiation, λ = 0.71073 Å
*a* = 11.07179 (16) Å	Cell parameters from 5641 reflections
*b* = 12.50386 (16) Å	θ = 2.5–45.2°
*c* = 7.53969 (11) Å	µ = 3.43 mm^−^^1^
β = 103.2349 (14)°	*T* = 293 K
*V* = 1016.07 (3) Å^3^	Block, metallic reddish red
*Z* = 4	0.12 × 0.08 × 0.07 mm

### Dipotassium nickel polyphosphate Data collection

**Table:**

SuperNova diffractometer	*R*_int_ = 0.035
θ/2θ scans	θ_max_ = 45.2°, θ_min_ = 2.5°
Absorption correction: multi-scan (CrysAlisPro; Rigaku OD, 2022)	*h* = −22→21
*T*_min_ = 0.509, *T*_max_ = 0.710	*k* = −24→24
33158 measured reflections	*l* = −14→14
8076 independent reflections	3 standard reflections every 120 min
7869 reflections with *I* > 2σ(*I*)	intensity decay: none

### Dipotassium nickel polyphosphate Refinement

**Table:**

Refinement on *F*^2^	2 restraints
Least-squares matrix: full	*w* = 1/[σ^2^(*F*_o_^2^) + (0.0564*P*)^2^ + 1.2215*P*] where *P* = (*F*_o_^2^ + 2*F*_c_^2^)/3
*R*[*F*^2^ > 2σ(*F*^2^)] = 0.031	(Δ/σ)_max_ = 0.001
*wR*(*F*^2^) = 0.088	Δρ_max_ = 0.83 e Å^−^^3^
*S* = 1.07	Δρ_min_ = −2.85 e Å^−^^3^
8076 reflections	Absolute structure: Refined as an inversion twin
173 parameters	Absolute structure parameter: 0.539 (9)

### Dipotassium nickel polyphosphate Special details

**Table:**

Geometry. All esds (except the esd in the dihedral angle between two l.s. planes) are estimated using the full covariance matrix. The cell esds are taken into account individually in the estimation of esds in distances, angles and torsion angles; correlations between esds in cell parameters are only used when they are defined by crystal symmetry. An approximate (isotropic) treatment of cell esds is used for estimating esds involving l.s. planes.
Refinement. Refined as a 2-component inversion twin.Data were collected on a SuperNova diffractometer equipped with a (Mo)X-ray Source and an Atlas CCD detector. Cell refinement and data reduction were performed with CrysAlisPro 1.171.42.49 (Rigaku, 2022) and adsorption correction withSCALE3 ABSPACK scaling algorithm (Rigaku Oxford Diffraction, 2022). Using the SHELX software package, the structure was solved by the direct method with the SHELXS program (Sheldrick, 2015a) and refined by the full-matrix least-squares method using SHELXL program (Sheldrick, 2015b). The programs *Ortep-3 for Windows* (Farrugia, 2012) and *DIAMOND* (Brandenburg, 2006) were used for molecular graphics and the software *publCIF* (Westrip, 2010) to prepare material for publication.

### Dipotassium nickel polyphosphate Fractional atomic coordinates and isotropic or equivalent isotropic displacement parameters (Å^2^)

**Table:**

	*x*	*y*	*z*	*U*_iso_*/*U*_eq_	
Ni1	0.94642 (3)	0.34669 (2)	0.79387 (4)	0.00586 (5)	
K1	0.61359 (4)	0.63341 (3)	0.12530 (6)	0.00747 (6)	
K2	1.31600 (5)	0.37393 (4)	0.96892 (7)	0.01014 (7)	
P1	1.05470 (5)	0.57511 (4)	0.70758 (7)	0.00330 (7)	
P2	0.83590 (5)	0.57688 (4)	0.86578 (7)	0.00340 (7)	
P3	0.62807 (5)	0.68963 (4)	0.62381 (7)	0.00407 (7)	
P4	1.28454 (5)	0.66069 (4)	0.97345 (7)	0.00384 (7)	
O1	0.94041 (16)	0.62429 (12)	0.7749 (2)	0.0065 (2)	
O2	0.84766 (14)	0.45926 (12)	0.8902 (2)	0.00540 (19)	
O3	0.71177 (15)	0.58966 (12)	0.7095 (2)	0.0066 (2)	
O4	1.16650 (16)	0.59314 (15)	0.8769 (2)	0.0088 (2)	
O5	1.04508 (15)	0.45704 (12)	0.6879 (2)	0.0058 (2)	
O6	0.82806 (16)	0.64783 (12)	1.0220 (2)	0.0059 (2)	
O7	1.07218 (15)	0.64267 (13)	0.5525 (2)	0.0059 (2)	
O8	1.31674 (15)	0.73723 (13)	0.8407 (2)	0.0074 (2)	
O9	1.23326 (16)	0.72198 (13)	1.1278 (2)	0.0070 (2)	
O10	1.37679 (15)	0.58280 (13)	1.0701 (2)	0.0085 (2)	
O11	0.55585 (16)	0.72302 (13)	0.7586 (2)	0.0076 (2)	
O12	0.56100 (16)	0.66049 (14)	0.4387 (2)	0.0085 (2)	

### Dipotassium nickel polyphosphate Atomic displacement parameters (Å^2^)

**Table:**

	*U*^11^	*U*^22^	*U*^33^	*U*^12^	*U*^13^	*U*^23^
Ni1	0.00581 (9)	0.00526 (8)	0.00660 (8)	0.00012 (7)	0.00160 (7)	0.00007 (7)
K1	0.00615 (13)	0.00922 (13)	0.00764 (13)	−0.00002 (12)	0.00283 (10)	−0.00036 (12)
K2	0.00994 (16)	0.00745 (14)	0.01295 (17)	−0.00041 (12)	0.00245 (13)	−0.00230 (12)
P1	0.00303 (16)	0.00325 (16)	0.00364 (15)	−0.00080 (12)	0.00081 (12)	−0.00030 (12)
P2	0.00332 (16)	0.00287 (15)	0.00425 (15)	0.00051 (12)	0.00139 (12)	−0.00014 (12)
P3	0.00380 (16)	0.00497 (16)	0.00343 (15)	0.00063 (12)	0.00079 (12)	0.00020 (13)
P4	0.00303 (16)	0.00393 (17)	0.00437 (16)	−0.00011 (12)	0.00048 (13)	−0.00040 (12)
O1	0.0059 (5)	0.0051 (4)	0.0101 (6)	0.0000 (4)	0.0052 (4)	0.0001 (4)
O2	0.0059 (5)	0.0030 (4)	0.0075 (5)	0.0009 (3)	0.0020 (4)	0.0005 (3)
O3	0.0050 (5)	0.0051 (4)	0.0085 (5)	0.0007 (4)	−0.0008 (4)	0.0003 (4)
O4	0.0073 (5)	0.0110 (6)	0.0063 (5)	−0.0048 (4)	−0.0023 (4)	0.0003 (4)
O5	0.0065 (5)	0.0037 (4)	0.0076 (5)	−0.0006 (4)	0.0026 (4)	−0.0007 (4)
O6	0.0069 (5)	0.0053 (5)	0.0057 (5)	0.0009 (4)	0.0019 (4)	−0.0014 (4)
O7	0.0065 (5)	0.0067 (5)	0.0048 (5)	−0.0015 (4)	0.0017 (4)	0.0008 (4)
O8	0.0073 (5)	0.0075 (5)	0.0082 (5)	−0.0011 (4)	0.0035 (4)	0.0013 (4)
O9	0.0087 (5)	0.0064 (5)	0.0064 (5)	0.0020 (4)	0.0029 (4)	−0.0011 (4)
O10	0.0060 (5)	0.0068 (5)	0.0112 (6)	0.0023 (4)	−0.0007 (4)	0.0016 (4)
O11	0.0085 (5)	0.0078 (5)	0.0078 (5)	0.0023 (4)	0.0043 (4)	0.0005 (4)
O12	0.0074 (5)	0.0129 (6)	0.0044 (5)	−0.0006 (4)	−0.0004 (4)	−0.0008 (4)

### Dipotassium nickel polyphosphate Geometric parameters (Å, º)

**Table:**

Ni1—K1^i^	3.6212 (6)	K2—O6^vi^	2.8540 (16)
Ni1—K1^ii^	3.8294 (5)	K2—O7^i^	2.9168 (18)
Ni1—K2^iii^	3.7520 (6)	K2—O8^i^	3.1274 (18)
Ni1—K2	4.0161 (6)	K2—O9^iv^	2.7951 (17)
Ni1—O2	2.0168 (15)	K2—O10^iv^	3.273 (2)
Ni1—O5	2.0335 (16)	K2—O10	2.7605 (18)
Ni1—O6^iv^	2.1666 (16)	K2—O11^x^	3.2612 (18)
Ni1—O7^i^	2.1253 (16)	K2—O12^x^	2.8069 (19)
Ni1—O8^v^	2.0717 (16)	P1—O1	1.5910 (17)
Ni1—O11^vi^	2.0194 (16)	P1—O4	1.5779 (16)
K1—P2^vii^	3.5486 (7)	P1—O5	1.4852 (16)
K1—P2^iv^	3.7705 (7)	P1—O7	1.4903 (16)
K1—P4^viii^	3.5763 (7)	P2—O1	1.5882 (17)
K1—O2^iv^	3.1108 (16)	P2—O2	1.4842 (16)
K1—O3^iv^	3.0080 (16)	P2—O3	1.6002 (16)
K1—O6^vii^	2.6693 (18)	P2—O6	1.4928 (16)
K1—O7^ix^	2.8697 (16)	P3—O3	1.6016 (16)
K1—O8^ix^	2.9396 (17)	P3—O9^ix^	1.6009 (17)
K1—O10^viii^	2.6358 (18)	P3—O11	1.4893 (17)
K1—O11^vii^	2.9154 (17)	P3—O12	1.4691 (17)
K1—O12	2.5817 (18)	P4—O4	1.5860 (17)
K2—P3^x^	3.4765 (7)	P4—O8	1.4858 (17)
K2—P4	3.6034 (7)	P4—O9	1.6022 (17)
K2—O4	3.195 (2)	P4—O10	1.4771 (16)
K2—O5	3.4141 (17)		
			
K1^i^—Ni1—K1^ii^	125.300 (12)	O10—K2—O5	90.43 (4)
K1^i^—Ni1—K2^iii^	67.218 (12)	O10—K2—O6^vi^	153.87 (5)
K1^ii^—Ni1—K2	62.662 (11)	O10—K2—O7^i^	100.77 (5)
K1^i^—Ni1—K2	171.099 (12)	O10—K2—O8^i^	102.85 (5)
K2^iii^—Ni1—K1^ii^	88.300 (11)	O10—K2—O9^iv^	131.91 (5)
K2^iii^—Ni1—K2	119.195 (13)	O10—K2—O10^iv^	90.52 (4)
O2—Ni1—K1^ii^	118.16 (5)	O10—K2—O11^x^	93.26 (5)
O2—Ni1—K1^i^	59.09 (5)	O10—K2—O12^x^	89.34 (5)
O2—Ni1—K2^iii^	126.08 (5)	O11^x^—K2—P3^x^	25.28 (3)
O2—Ni1—K2	114.71 (5)	O11^x^—K2—P4	115.41 (3)
O2—Ni1—O5	92.99 (6)	O11^x^—K2—O5	173.59 (4)
O2—Ni1—O6^iv^	93.34 (6)	O11^x^—K2—O10^iv^	111.68 (4)
O2—Ni1—O7^i^	85.48 (6)	O12^x^—K2—P3^x^	24.16 (3)
O2—Ni1—O8^v^	86.88 (7)	O12^x^—K2—P4	104.51 (4)
O2—Ni1—O11^vi^	166.65 (7)	O12^x^—K2—O4	125.18 (5)
O5—Ni1—K1^ii^	120.40 (5)	O12^x^—K2—O5	136.41 (5)
O5—Ni1—K1^i^	114.24 (5)	O12^x^—K2—O6^vi^	81.05 (5)
O5—Ni1—K2	58.20 (5)	O12^x^—K2—O7^i^	165.05 (5)
O5—Ni1—K2^iii^	113.79 (5)	O12^x^—K2—O8^i^	101.57 (5)
O5—Ni1—O6^iv^	82.52 (6)	O12^x^—K2—O10^iv^	62.93 (5)
O5—Ni1—O7^i^	91.15 (6)	O12^x^—K2—O11^x^	48.97 (4)
O5—Ni1—O8^v^	166.15 (7)	K1^xi^—P1—K2^iv^	65.244 (13)
O6^iv^—Ni1—K1^i^	47.10 (5)	O1—P1—K1^xi^	81.92 (6)
O6^iv^—Ni1—K1^ii^	137.45 (4)	O1—P1—K2^iv^	146.61 (6)
O6^iv^—Ni1—K2	131.17 (5)	O4—P1—K1^xi^	82.08 (7)
O6^iv^—Ni1—K2^iii^	49.19 (4)	O4—P1—K2^iv^	79.50 (7)
O7^i^—Ni1—K1^ii^	47.74 (4)	O4—P1—O1	102.73 (10)
O7^i^—Ni1—K1^i^	135.89 (5)	O5—P1—K1^xi^	162.46 (7)
O7^i^—Ni1—K2^iii^	135.94 (4)	O5—P1—K2^iv^	99.47 (7)
O7^i^—Ni1—K2	44.68 (5)	O5—P1—O1	111.84 (9)
O7^i^—Ni1—O6^iv^	173.50 (6)	O5—P1—O4	104.35 (10)
O8^v^—Ni1—K1^i^	54.26 (5)	O5—P1—O7	120.29 (9)
O8^v^—Ni1—K1^ii^	71.27 (5)	O7—P1—K1^xi^	42.80 (6)
O8^v^—Ni1—K2^iii^	56.46 (5)	O7—P1—K2^iv^	44.18 (7)
O8^v^—Ni1—K2	133.93 (5)	O7—P1—O1	106.67 (9)
O8^v^—Ni1—O6^iv^	83.66 (6)	O7—P1—O4	109.57 (10)
O8^v^—Ni1—O7^i^	102.64 (7)	K1^xii^—P2—K1^i^	86.856 (17)
O11^vi^—Ni1—K1^ii^	48.53 (5)	K1^xii^—P2—K2^xiii^	67.271 (14)
O11^vi^—Ni1—K1^i^	126.83 (5)	K1^i^—P2—K2^xiii^	135.710 (18)
O11^vi^—Ni1—K2^iii^	60.25 (5)	O1—P2—K1^xii^	146.03 (6)
O11^vi^—Ni1—K2	60.75 (5)	O1—P2—K1^i^	120.85 (7)
O11^vi^—Ni1—O5	94.32 (7)	O1—P2—K2^xiii^	78.82 (6)
O11^vi^—Ni1—O6^iv^	98.67 (7)	O1—P2—O3	103.48 (9)
O11^vi^—Ni1—O7^i^	83.22 (7)	O2—P2—K1^xii^	100.44 (7)
O11^vi^—Ni1—O8^v^	88.67 (7)	O2—P2—K1^i^	52.96 (6)
Ni1^iv^—K1—P2^iv^	51.633 (11)	O2—P2—K2^xiii^	161.42 (7)
P2^vii^—K1—Ni1^iv^	55.151 (12)	O2—P2—O1	111.80 (9)
P2^vii^—K1—P2^iv^	70.573 (13)	O2—P2—O3	103.08 (9)
P2^vii^—K1—P4^viii^	128.817 (18)	O2—P2—O6	120.69 (9)
P4^viii^—K1—Ni1^iv^	170.418 (17)	O3—P2—K1^i^	50.17 (6)
P4^viii^—K1—P2^iv^	136.511 (17)	O3—P2—K1^xii^	78.39 (7)
O2^iv^—K1—Ni1^iv^	33.80 (3)	O3—P2—K2^xiii^	88.45 (6)
O2^iv^—K1—P2^iv^	22.39 (3)	O6—P2—K1^i^	130.12 (7)
O2^iv^—K1—P2^vii^	72.46 (3)	O6—P2—K1^xii^	43.42 (7)
O2^iv^—K1—P4^viii^	151.52 (3)	O6—P2—K2^xiii^	40.83 (6)
O3^iv^—K1—Ni1^iv^	72.55 (3)	O6—P2—O1	107.34 (9)
O3^iv^—K1—P2^vii^	70.67 (4)	O6—P2—O3	109.04 (9)
O3^iv^—K1—P2^iv^	24.11 (3)	K2^xiii^—P3—K1	134.104 (18)
O3^iv^—K1—P4^viii^	116.65 (3)	K2^xiv^—P3—K1	79.126 (15)
O3^iv^—K1—O2^iv^	46.48 (4)	K2^xiv^—P3—K2^xiii^	136.71 (2)
O6^vii^—K1—Ni1^iv^	36.48 (4)	O3—P3—K1	98.32 (7)
O6^vii^—K1—P2^iv^	70.19 (4)	O3—P3—K2^xiv^	113.32 (6)
O6^vii^—K1—P2^vii^	22.61 (3)	O3—P3—K2^xiii^	91.25 (6)
O6^vii^—K1—P4^viii^	144.02 (4)	O9^ix^—P3—K1	90.70 (6)
O6^vii^—K1—O2^iv^	63.03 (5)	O9^ix^—P3—K2^xiii^	43.41 (6)
O6^vii^—K1—O3^iv^	79.73 (5)	O9^ix^—P3—K2^xiv^	145.79 (6)
O6^vii^—K1—O7^ix^	89.55 (5)	O9^ix^—P3—O3	100.37 (9)
O6^vii^—K1—O8^ix^	60.32 (5)	O11—P3—K1	145.19 (7)
O6^vii^—K1—O11^vii^	73.37 (5)	O11—P3—K2^xiii^	69.63 (7)
O7^ix^—K1—Ni1^iv^	96.04 (4)	O11—P3—K2^xiv^	69.25 (7)
O7^ix^—K1—P2^iv^	146.15 (4)	O11—P3—O3	107.15 (10)
O7^ix^—K1—P2^vii^	100.93 (4)	O11—P3—O9^ix^	107.19 (10)
O7^ix^—K1—P4^viii^	74.89 (4)	O12—P3—K1	27.68 (7)
O7^ix^—K1—O2^iv^	124.00 (4)	O12—P3—K2^xiv^	51.45 (8)
O7^ix^—K1—O3^iv^	168.30 (5)	O12—P3—K2^xiii^	153.56 (7)
O7^ix^—K1—O8^ix^	68.73 (5)	O12—P3—O3	108.05 (9)
O7^ix^—K1—O11^vii^	56.84 (5)	O12—P3—O9^ix^	113.35 (10)
O8^ix^—K1—Ni1^iv^	34.89 (3)	O12—P3—O11	118.97 (11)
O8^ix^—K1—P2^iv^	77.60 (3)	K1^xv^—P4—K1^xi^	126.000 (18)
O8^ix^—K1—P2^vii^	82.80 (3)	K1^xv^—P4—K2^i^	79.159 (15)
O8^ix^—K1—P4^viii^	135.97 (4)	K1^xv^—P4—K2	79.311 (16)
O8^ix^—K1—O2^iv^	55.29 (4)	K2—P4—K1^xi^	133.819 (19)
O8^ix^—K1—O3^iv^	101.52 (5)	K2^i^—P4—K1^xi^	133.235 (18)
O10^viii^—K1—Ni1^iv^	165.34 (4)	K2—P4—K2^i^	84.614 (14)
O10^viii^—K1—P2^iv^	115.13 (4)	O4—P4—K1^xi^	79.54 (7)
O10^viii^—K1—P2^vii^	130.96 (4)	O4—P4—K1^xv^	140.19 (8)
O10^viii^—K1—P4^viii^	21.38 (4)	O4—P4—K2	62.42 (7)
O10^viii^—K1—O2^iv^	131.63 (5)	O4—P4—K2^i^	106.16 (7)
O10^viii^—K1—O3^iv^	96.26 (5)	O4—P4—O9	101.31 (10)
O10^viii^—K1—O6^vii^	152.78 (6)	O8—P4—K1^xi^	42.82 (7)
O10^viii^—K1—O7^ix^	95.42 (5)	O8—P4—K1^xv^	83.87 (7)
O10^viii^—K1—O8^ix^	145.79 (5)	O8—P4—K2	126.58 (7)
O10^viii^—K1—O11^vii^	87.03 (5)	O8—P4—K2^i^	140.74 (7)
O11^vii^—K1—Ni1^iv^	106.92 (4)	O8—P4—O4	109.49 (10)
O11^vii^—K1—P2^iv^	134.53 (4)	O8—P4—O9	111.14 (9)
O11^vii^—K1—P2^vii^	65.34 (3)	O9—P4—K1^xi^	88.55 (6)
O11^vii^—K1—P4^viii^	70.95 (4)	O9—P4—K1^xv^	108.51 (6)
O11^vii^—K1—O2^iv^	136.18 (5)	O9—P4—K2^i^	44.69 (6)
O11^vii^—K1—O3^iv^	123.15 (5)	O9—P4—K2	122.27 (6)
O11^vii^—K1—O8^ix^	106.65 (5)	O10—P4—K1^xv^	40.58 (7)
O12—K1—Ni1^iv^	95.42 (4)	O10—P4—K1^xi^	162.48 (8)
O12—K1—P2^vii^	149.42 (4)	O10—P4—K2^i^	61.96 (8)
O12—K1—P2^iv^	84.87 (4)	O10—P4—K2	44.86 (7)
O12—K1—P4^viii^	81.60 (4)	O10—P4—O4	106.22 (10)
O12—K1—O2^iv^	77.92 (5)	O10—P4—O8	120.76 (10)
O12—K1—O3^iv^	94.28 (5)	O10—P4—O9	106.09 (10)
O12—K1—O6^vii^	131.32 (5)	P2—O1—P1	134.80 (10)
O12—K1—O7^ix^	89.27 (5)	Ni1—O2—K1^i^	87.11 (5)
O12—K1—O8^ix^	74.13 (5)	P2—O2—Ni1	133.34 (10)
O12—K1—O10^viii^	75.61 (6)	P2—O2—K1^i^	104.66 (7)
O12—K1—O11^vii^	140.45 (5)	P2—O3—K1^i^	105.71 (7)
P3^x^—K2—P4	108.365 (18)	P2—O3—P3	134.04 (11)
O4—K2—P3^x^	134.00 (3)	P3—O3—K1^i^	119.39 (8)
O4—K2—P4	26.10 (3)	P1—O4—K2	108.94 (9)
O4—K2—O5	42.80 (4)	P1—O4—P4	149.18 (13)
O4—K2—O10^iv^	82.19 (5)	P4—O4—K2	91.47 (8)
O4—K2—O11^x^	140.01 (4)	Ni1—O5—K2	91.39 (5)
O5—K2—P3^x^	160.47 (3)	P1—O5—Ni1	131.91 (10)
O5—K2—P4	68.28 (3)	P1—O5—K2	102.00 (7)
O6^vi^—K2—P3^x^	73.47 (4)	Ni1^i^—O6—K1^xii^	96.42 (6)
O6^vi^—K2—P4	170.35 (4)	Ni1^i^—O6—K2^xiii^	95.74 (5)
O6^vi^—K2—O4	152.31 (5)	K1^xii^—O6—K2^xiii^	95.26 (5)
O6^vi^—K2—O5	113.25 (4)	P2—O6—Ni1^i^	129.30 (10)
O6^vi^—K2—O7^i^	85.13 (5)	P2—O6—K1^xii^	113.98 (9)
O6^vi^—K2—O8^i^	56.20 (5)	P2—O6—K2^xiii^	119.17 (9)
O6^vi^—K2—O10^iv^	106.24 (5)	Ni1^iv^—O7—K1^xi^	99.02 (6)
O6^vi^—K2—O11^x^	62.17 (5)	Ni1^iv^—O7—K2^iv^	104.50 (6)
O7^i^—K2—P3^x^	143.99 (4)	K1^xi^—O7—K2^iv^	89.74 (5)
O7^i^—K2—P4	88.43 (3)	P1—O7—Ni1^iv^	125.44 (10)
O7^i^—K2—O4	69.44 (5)	P1—O7—K1^xi^	116.54 (8)
O7^i^—K2—O5	55.30 (4)	P1—O7—K2^iv^	114.96 (9)
O7^i^—K2—O8^i^	65.62 (5)	Ni1^xvi^—O8—K1^xi^	90.84 (6)
O7^i^—K2—O10^iv^	127.26 (5)	Ni1^xvi^—O8—K2^iv^	90.02 (6)
O7^i^—K2—O11^x^	118.74 (5)	K1^xi^—O8—K2^iv^	84.52 (4)
O8^i^—K2—P3^x^	78.40 (3)	P4—O8—Ni1^xvi^	145.38 (11)
O8^i^—K2—P4	114.47 (3)	P4—O8—K1^xi^	117.09 (9)
O8^i^—K2—O4	118.08 (5)	P4—O8—K2^iv^	111.44 (8)
O8^i^—K2—O5	120.88 (4)	P3^xi^—O9—K2^i^	113.41 (8)
O8^i^—K2—O10^iv^	159.73 (5)	P3^xi^—O9—P4	133.93 (11)
O8^i^—K2—O11^x^	53.12 (4)	P4—O9—K2^i^	111.54 (8)
O9^iv^—K2—P3^x^	107.59 (4)	K1^xv^—O10—K2	116.20 (6)
O9^iv^—K2—P4	115.06 (4)	K1^xv^—O10—K2^i^	102.79 (5)
O9^iv^—K2—O4	97.12 (5)	K2—O10—K2^i^	108.77 (6)
O9^iv^—K2—O5	60.22 (4)	P4—O10—K1^xv^	118.04 (9)
O9^iv^—K2—O6^vi^	72.48 (5)	P4—O10—K2^i^	94.57 (8)
O9^iv^—K2—O7^i^	92.51 (5)	P4—O10—K2	112.97 (9)
O9^iv^—K2—O8^i^	124.55 (5)	Ni1^xiii^—O11—K1^xii^	100.21 (6)
O9^iv^—K2—O10^iv^	47.05 (4)	Ni1^xiii^—O11—K2^xiv^	87.23 (6)
O9^iv^—K2—O11^x^	120.12 (5)	K1^xii^—O11—K2^xiv^	117.41 (6)
O9^iv^—K2—O12^x^	88.78 (5)	P3—O11—Ni1^xiii^	137.44 (11)
O10^iv^—K2—P3^x^	87.07 (3)	P3—O11—K1^xii^	120.26 (9)
O10—K2—P3^x^	87.87 (4)	P3—O11—K2^xiv^	85.46 (7)
O10^iv^—K2—P4	83.39 (3)	K1—O12—K2^xiv^	118.61 (6)
O10—K2—P4	22.17 (3)	P3—O12—K1	136.99 (11)
O10—K2—O4	47.91 (4)	P3—O12—K2^xiv^	104.38 (9)
O10^iv^—K2—O5	73.49 (4)		
			
K1^xi^—P1—O1—P2	−175.17 (16)	O1—P2—O2—Ni1	−12.87 (16)
K1^xi^—P1—O4—K2	−149.54 (7)	O1—P2—O2—K1^i^	−112.96 (8)
K1^xi^—P1—O4—P4	−20.6 (3)	O1—P2—O3—K1^i^	119.13 (8)
K1^xi^—P1—O5—Ni1	−159.81 (12)	O1—P2—O3—P3	−72.00 (17)
K1^xi^—P1—O5—K2	97.2 (2)	O1—P2—O6—Ni1^i^	−79.45 (13)
K1^xi^—P1—O7—Ni1^iv^	−124.79 (16)	O1—P2—O6—K1^xii^	159.07 (8)
K1^xi^—P1—O7—K2^iv^	103.12 (10)	O1—P2—O6—K2^xiii^	47.77 (11)
K1^i^—P2—O1—P1	−59.95 (18)	O2—P2—O1—P1	−1.07 (19)
K1^xii^—P2—O1—P1	159.50 (8)	O2—P2—O3—K1^i^	2.53 (10)
K1^xii^—P2—O2—Ni1	178.03 (11)	O2—P2—O3—P3	171.40 (15)
K1^i^—P2—O2—Ni1	100.09 (14)	O2—P2—O6—Ni1^i^	50.18 (16)
K1^xii^—P2—O2—K1^i^	77.94 (5)	O2—P2—O6—K1^xii^	−71.30 (12)
K1^xii^—P2—O3—K1^i^	−95.65 (6)	O2—P2—O6—K2^xiii^	177.41 (8)
K1^i^—P2—O3—P3	168.9 (2)	O3—P2—O1—P1	−111.34 (16)
K1^xii^—P2—O3—P3	73.22 (15)	O3—P2—O2—Ni1	97.66 (13)
K1^xii^—P2—O6—Ni1^i^	121.48 (15)	O3—P2—O2—K1^i^	−2.43 (10)
K1^i^—P2—O6—Ni1^i^	115.61 (10)	O3—P2—O6—Ni1^i^	169.07 (11)
K1^i^—P2—O6—K1^xii^	−5.87 (12)	O3—P2—O6—K1^xii^	47.60 (11)
K1^xii^—P2—O6—K2^xiii^	−111.29 (12)	O3—P2—O6—K2^xiii^	−63.70 (11)
K1^i^—P2—O6—K2^xiii^	−117.17 (7)	O3—P3—O11—Ni1^xiii^	170.05 (14)
K1—P3—O3—K1^i^	−63.59 (8)	O3—P3—O11—K1^xii^	10.20 (12)
K1—P3—O3—P2	128.73 (15)	O3—P3—O11—K2^xiv^	−109.04 (7)
K1—P3—O11—Ni1^xiii^	−54.8 (2)	O3—P3—O12—K1	−72.92 (16)
K1—P3—O11—K1^xii^	145.33 (7)	O3—P3—O12—K2^xiv^	105.83 (9)
K1—P3—O11—K2^xiv^	26.10 (12)	O4—P1—O1—P2	−95.24 (17)
K1—P3—O12—K2^xiv^	178.75 (19)	O4—P1—O5—Ni1	90.47 (14)
K1^xi^—P4—O4—K2	152.97 (5)	O4—P1—O5—K2	−12.48 (10)
K1^xv^—P4—O4—K2	17.57 (12)	O4—P1—O7—Ni1^iv^	−177.97 (11)
K1^xv^—P4—O4—P1	−115.0 (2)	O4—P1—O7—K1^xi^	−53.18 (12)
K1^xi^—P4—O4—P1	20.4 (3)	O4—P1—O7—K2^iv^	49.94 (12)
K1^xi^—P4—O8—Ni1^xvi^	140.4 (2)	O4—P4—O8—Ni1^xvi^	−171.34 (17)
K1^xv^—P4—O8—Ni1^xvi^	−29.95 (18)	O4—P4—O8—K1^xi^	48.30 (12)
K1^xv^—P4—O8—K1^xi^	−170.31 (8)	O4—P4—O8—K2^iv^	−46.62 (12)
K1^xi^—P4—O8—K2^iv^	−94.92 (10)	O4—P4—O9—K2^i^	101.62 (9)
K1^xv^—P4—O8—K2^iv^	94.78 (6)	O4—P4—O9—P3^xi^	−65.16 (17)
K1^xi^—P4—O9—K2^i^	−179.35 (6)	O4—P4—O10—K1^xv^	152.89 (10)
K1^xv^—P4—O9—K2^i^	−51.64 (8)	O4—P4—O10—K2	12.62 (13)
K1^xv^—P4—O9—P3^xi^	141.58 (13)	O4—P4—O10—K2^i^	−99.99 (9)
K1^xi^—P4—O9—P3^xi^	13.87 (15)	O5—P1—O1—P2	16.13 (19)
K1^xi^—P4—O10—K1^xv^	45.8 (3)	O5—P1—O4—K2	13.82 (11)
K1^xi^—P4—O10—K2^i^	153.0 (2)	O5—P1—O4—P4	142.7 (3)
K1^xv^—P4—O10—K2	−140.27 (15)	O5—P1—O7—Ni1^iv^	61.21 (15)
K1^xi^—P4—O10—K2	−94.4 (2)	O5—P1—O7—K1^xi^	−174.00 (8)
K1^xv^—P4—O10—K2^i^	107.12 (10)	O5—P1—O7—K2^iv^	−70.88 (11)
K2^iv^—P1—O1—P2	174.58 (7)	O6—P2—O1—P1	133.43 (16)
K2^iv^—P1—O4—K2	−83.38 (6)	O6—P2—O2—Ni1	−140.52 (12)
K2^iv^—P1—O4—P4	45.5 (3)	O6—P2—O2—K1^i^	119.39 (9)
K2^iv^—P1—O5—Ni1	171.95 (10)	O6—P2—O3—K1^i^	−126.86 (8)
K2^iv^—P1—O5—K2	69.00 (5)	O6—P2—O3—P3	42.01 (19)
K2^iv^—P1—O7—Ni1^iv^	132.09 (15)	O7—P1—O1—P2	149.54 (15)
K2^iv^—P1—O7—K1^xi^	−103.12 (10)	O7—P1—O4—K2	−116.23 (9)
K2^xiii^—P2—O1—P1	163.00 (16)	O7—P1—O4—P4	12.7 (3)
K2^xiii^—P2—O2—Ni1	−135.19 (15)	O7—P1—O5—Ni1	−146.17 (12)
K2^xiii^—P2—O2—K1^i^	124.72 (18)	O7—P1—O5—K2	110.88 (9)
K2^xiii^—P2—O3—K1^i^	−162.76 (6)	O8—P4—O4—K2	121.90 (8)
K2^xiii^—P2—O3—P3	6.11 (15)	O8—P4—O4—P1	−10.7 (3)
K2^xiii^—P2—O6—Ni1^i^	−127.23 (17)	O8—P4—O9—K2^i^	−142.14 (9)
K2^xiii^—P2—O6—K1^xii^	111.29 (12)	O8—P4—O9—P3^xi^	51.08 (18)
K2^xiii^—P3—O3—K1^i^	161.44 (8)	O8—P4—O10—K1^xv^	27.62 (15)
K2^xiv^—P3—O3—K1^i^	18.10 (11)	O8—P4—O10—K2	−112.65 (11)
K2^xiii^—P3—O3—P2	−6.24 (16)	O8—P4—O10—K2^i^	134.73 (9)
K2^xiv^—P3—O3—P2	−149.58 (13)	O9^ix^—P3—O3—K1^i^	−155.84 (9)
K2^xiii^—P3—O11—Ni1^xiii^	85.23 (15)	O9^ix^—P3—O3—P2	36.48 (18)
K2^xiv^—P3—O11—Ni1^xiii^	−80.91 (15)	O9^ix^—P3—O11—Ni1^xiii^	63.04 (18)
K2^xiii^—P3—O11—K1^xii^	−74.63 (8)	O9^ix^—P3—O11—K1^xii^	−96.82 (10)
K2^xiv^—P3—O11—K1^xii^	119.23 (10)	O9^ix^—P3—O11—K2^xiv^	143.95 (7)
K2^xiii^—P3—O11—K2^xiv^	166.14 (5)	O9^ix^—P3—O12—K1	37.38 (18)
K2^xiii^—P3—O12—K1	62.0 (2)	O9^ix^—P3—O12—K2^xiv^	−143.87 (8)
K2^xiv^—P3—O12—K1	−178.75 (19)	O9—P4—O4—K2	−120.65 (7)
K2^xiii^—P3—O12—K2^xiv^	−119.20 (14)	O9—P4—O4—P1	106.8 (3)
K2^i^—P4—O4—K2	−74.83 (5)	O9—P4—O8—Ni1^xvi^	77.6 (2)
K2—P4—O4—P1	−132.6 (3)	O9—P4—O8—K1^xi^	−62.80 (11)
K2^i^—P4—O4—P1	152.6 (3)	O9—P4—O8—K2^iv^	−157.72 (8)
K2^i^—P4—O8—Ni1^xvi^	34.6 (3)	O9—P4—O10—K1^xv^	−99.86 (11)
K2—P4—O8—Ni1^xvi^	−101.76 (18)	O9—P4—O10—K2	119.87 (9)
K2—P4—O8—K1^xi^	117.88 (7)	O9—P4—O10—K2^i^	7.26 (9)
K2^i^—P4—O8—K1^xi^	−105.80 (10)	O10—P4—O4—K2	−10.01 (10)
K2—P4—O8—K2^iv^	22.96 (11)	O10—P4—O4—P1	−142.6 (3)
K2^i^—P4—O8—K2^iv^	159.29 (5)	O10—P4—O8—Ni1^xvi^	−47.6 (2)
K2—P4—O9—K2^i^	37.22 (10)	O10—P4—O8—K1^xi^	172.04 (9)
K2—P4—O9—P3^xi^	−129.56 (12)	O10—P4—O8—K2^iv^	77.12 (12)
K2^i^—P4—O9—P3^xi^	−166.8 (2)	O10—P4—O9—K2^i^	−9.12 (11)
K2^i^—P4—O10—K1^xv^	−107.12 (10)	O10—P4—O9—P3^xi^	−175.90 (14)
K2—P4—O10—K1^xv^	140.27 (15)	O11—P3—O3—K1^i^	92.39 (11)
K2—P4—O10—K2^i^	−112.61 (10)	O11—P3—O3—P2	−75.29 (18)
K2^i^—P4—O10—K2	112.61 (10)	O11—P3—O12—K1	164.75 (12)
O1—P1—O4—K2	130.66 (8)	O11—P3—O12—K2^xiv^	−16.50 (12)
O1—P1—O4—P4	−100.4 (3)	O12—P3—O3—K1^i^	−36.92 (13)
O1—P1—O5—Ni1	−19.87 (16)	O12—P3—O3—P2	155.39 (15)
O1—P1—O5—K2	−122.82 (8)	O12—P3—O11—Ni1^xiii^	−67.17 (19)
O1—P1—O7—Ni1^iv^	−67.44 (13)	O12—P3—O11—K1^xii^	132.98 (10)
O1—P1—O7—K1^xi^	57.35 (10)	O12—P3—O11—K2^xiv^	13.74 (10)
O1—P1—O7—K2^iv^	160.47 (8)		

Symmetry codes: (i) *x*, −*y*+1, *z*+1/2; (ii) *x*+1/2, *y*−1/2, *z*+1; (iii) *x*−1/2, −*y*+1/2, *z*−1/2; (iv) *x*, −*y*+1, *z*−1/2; (v) *x*−1/2, *y*−1/2, *z*; (vi) *x*+1/2, *y*−1/2, *z*; (vii) *x*, *y*, *z*−1; (viii) *x*−1, *y*, *z*−1; (ix) *x*−1/2, −*y*+3/2, *z*−1/2; (x) *x*+1, −*y*+1, *z*+1/2; (xi) *x*+1/2, −*y*+3/2, *z*+1/2; (xii) *x*, *y*, *z*+1; (xiii) *x*−1/2, *y*+1/2, *z*; (xiv) *x*−1, −*y*+1, *z*−1/2; (xv) *x*+1, *y*, *z*+1; (xvi) *x*+1/2, *y*+1/2, *z*.
